# Mobilising knowledge between practitioners and researchers to iteratively refine a complex intervention (DAFNE*plus*) pre-trial: protocol for a structured, collaborative working group process

**DOI:** 10.1186/s40814-018-0314-4

**Published:** 2018-07-03

**Authors:** Jenna P. Breckenridge, Carla Gianfrancesco, Nicole de Zoysa, Julia Lawton, David Rankin, Elizabeth Coates

**Affiliations:** 1 School of Nursing and Health Sciences, University of Dundee, 11 Airlie Place, Dundee, DD1 4HJ UK; 2 Sheffield Diabetes and Endocrine Centre, Sheffield Teaching Hospitals NHS Foundation Trust, Northern General Hospital, Herries Road, Sheffield, S5 7AU UK; 3 Diabetes Centre, King’s College Hospital, Denmark Hill, London, SE5 9RS UK; 4 The Usher Institute of Population Health Sciences and Informatics, Edinburgh Medical School of Molecular, Genetic and Population Health Sciences, Teviot Place, Edinburgh, EH8 9AG UK; 5 School of Health and Related Research (ScHARR), University of Sheffield, Regent Court, 30 Regent Street, Sheffield, S1 4DA UK

**Keywords:** Knowledge mobilisation, Intervention development, Process evaluation, Qualitative research, Type 1 diabetes, Pilot study, Feasibility study, Collaborative working, Co-design

## Abstract

**Background:**

Randomised controlled trials (RCTs) of complex interventions often begin with a pilot phase to test the proposed methods and refine the intervention before it is trialled. Although the Medical Research Council (MRC) recommends regular communication between the practitioners delivering the intervention and the researchers evaluating it during the pilot phase, there is a lack of practical guidance about how to undertake this aspect of pre-trial work. This paper describes a novel structured process for collaborative working, which we developed to iteratively refine a complex intervention prior to an RCT. We also describe an in-built qualitative study to learn lessons about how this approach could be used by future study teams.

**Methods:**

This work forms part of a broader research programme to develop and trial a complex intervention for people with type 1 diabetes, called DAFNE*plus*. The intervention is being piloted in three National Health Service (NHS) diabetes centres in two waves, with refinements being incrementally implemented between each wave in response to real-time, collective learning (combining practitioner experience, process evaluation data and patient and public involvement via an advisory group). A structured ‘Collaborative Working Group’ (CWG) process, comprising monthly teleconferences and four strategically timed face-to-face meetings, is being used to identify and respond systematically to emerging implementation challenges and research findings. The group involves 25 members of the study team, including the multi-disciplinary practitioners delivering the intervention, the research teams conducting the process evaluation, the study manager and Chief Investigator. An in-built qualitative study comprising documentary analysis of meeting materials, discourse analysis of meeting transcripts, reflexive note taking, and thematic analysis of focus groups and interviews with CWG members is being undertaken to explore how the CWG works and how its processes and procedures might be improved.

**Discussion:**

The CWG process offers a potential model for collaborative working in future pre-trial pilot phases and intervention development studies that operationalises MRC guidance to progressively develop a complex intervention and foster shared ownership through genuine collaboration. The findings from the qualitative study will provide insight into how to best support collaborative working to achieve optimal intervention design.

## Background

Randomised controlled trials (RCTs) of complex interventions often begin with a pilot phase to increase the chances of developing an effective intervention and optimising the proposed trial design. During the pilot phase, the Medical Research Council (MRC) guidance recommends that a process evaluation is conducted in order to establish the feasibility and acceptability of the new intervention, ascertain the fidelity with which the intervention is delivered, explore the barriers to intervention delivery and identify any necessary refinements pre-trial [1]. Process evaluations are also used during the pilot phase to inform the intervention logic model and methodological choices for the process evaluation in the RCT [1]. Progressive intervention refinement calls for regular, real-time communication between the practitioners delivering the pilot intervention and the researchers evaluating it. While the MRC have emphasised the importance of agreeing iterative communication practices from the outset, there is a lack of operational guidance about how this communication should take place and how multi-disciplinary project teams make useful, timely, research-informed refinements to the intervention prior to implementation in an RCT. In this paper, we describe the protocol for a novel, structured process for collaborative working, which we have developed to iteratively refine a complex intervention (DAFNE*plus*) pre-trial. We also describe an in-built qualitative study to explore if, how and why this process works in order to provide insight and guidance for future teams wishing to use our collaborative working approach both in the pre-trial stage and in intervention development studies.

### The “DAFNE*plus*” research programme

The work presented here is part of an NIHR-funded programme grant to develop and trial a complex intervention for people with type 1 diabetes, called DAFNE*plus* (RP-PG-0514-20013). A description of DAFNE*plus* and its development is presented in Table 1.

**Table 1:** The origins and development of the DAFNE*plus* research programme

The DAFNE*plus* programme originates from the DAFNE (Dose Adjustment for Normal Eating) 5-day structured education programme for people with type 1 diabetes, which is widely delivered as part of routine clinical care in the UK and other countries [15, 16]. The current DAFNE programme is delivered by diabetes specialist nurses, dietitians and physicians and provides adults with type 1 diabetes training in flexible intensive insulin therapy to enable them to adjust their insulin doses and improve blood glucose control. People attending DAFNE courses are: (1) Taught how to count carbohydrates and, using ratios, to calculate mealtime insulin dose requirements relative to the amounts of carbohydrate ingested; (2) Advised to undertake regular review of blood glucose data and how to interpret patterns or changes in readings to inform adjustments to mealtime ratios and basal insulin doses to ensure readings are maintained within clinically recommended ranges; and (3) Instructed how to calculate and use corrective doses of insulin or additional carbohydrate to maintain blood glucose readings within recommended target ranges. Following their courses, participants are invited to attend an optional group follow-up session 6–8 weeks later before returning to attend routine clinical appointments every 6–12 months. An RCT to evaluate DAFNE established the importance of providing structured education to teach diabetes self-management skills to enable people with type 1 diabetes to better manage the disease [17]. However, although DAFNE has been shown to help people initially improve their glycaemic control, many are unable to sustain these improvements and control their blood glucose levels consistently over time [18]. A programme of research funded by the National Institute for Health Research (NIHR) was undertaken to investigate why people struggle over time and identified that DAFNE courses often fail to help participants sustain using new skills as part of their everyday lives. This research also highlighted a need to integrate within the DAFNE curriculum both support for behaviour change and structured health professional support, utilising new technologies to assist self-care [15]. In response, a multi-disciplinary research group was established and awarded funding by the NIHR for a new programme grant to develop the DAFNE*plus* intervention and enhance support provided to participants. This group used findings from the previous DAFNE studies, undertook a systematic review of the literature on structured education programmes and sought input from clinical and health psychologists with expertise in behaviour change to establish three inter-linked work packages to: (1) Modify the existing DAFNE curriculum and incorporate techniques for initiating and sustaining behaviour changes; (2) Develop structured follow-up support; and (3) Develop and assess how digital information communication technologies can be incorporated within the revised intervention to support health behaviours needed to optimise self-management of type 1 diabetes. The outputs from these projects have been combined and comprise the DAFNE*plus* intervention which aims to support and motivate participants to manage type 1 diabetes as part of their everyday lives in the longer term. The DAFNE*plus* pilot study is currently underway to refine each element of the intervention (curriculum, follow-up support and new technologies). This will be followed by a definitive pragmatic cluster RCT of DAFNE*plus* versus standard DAFNE in 14 DAFNE centres across the UK, due to commence in Autumn 2018.	

**Fig. 1:**
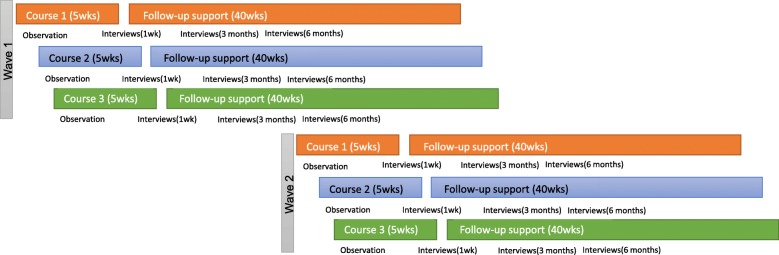
Overview of the DAFNE*plus* pilot study with embedded process evaluation. Legend: the coloured lines denote each of the three different study sites and show how intervention delivery is staggered in order to permit cross-learning at key time points. The text underneath each line identifies data collection points for the embedded process evaluation

The DAFNE*plus* pilot study has been granted a National Health Service Research Ethics Committee approval (16-WS-0230) and will take place in three sites, over two waves, with an embedded process evaluation. This process evaluation will involve observation of the course delivery, interviews with practitioners approximately 1 week after the course and longitudinal interviews with DAFNE*plus* participants at three key time points (approximately 1 week after the course, then at 3 and 6 months after course completion). Piloting the intervention in two waves is intended to optimise the design of the intervention by providing an opportunity to ‘try out’ and reflect on changes made to the intervention after the first pilot phase and before making definitive decisions about the final trial intervention. As such, knowledge generated from the first wave (combining practitioner experience, process evaluation and patient advisory group input) will be used to inform changes to the intervention in the second wave, before using knowledge generated in the second wave to inform further refinements prior to the RCT. Figure 1 provides a diagrammatic overview of the DAFNE*plus* pilot process.

## Methods/design

To support effective collaborative working between the practitioners and researchers on the DAFNE*plus* pilot study, we designed a novel structured process for iteratively sharing knowledge during and after each wave of the pilot phase to make progressive refinements to the intervention pre-trial. Here, we present the protocol for this ‘Collaborative Working Group’ (CWG) process comprising four strategically timed face-to-face meetings to identify, agree and operationalise changes to the intervention, and monthly teleconferences to permit real-time knowledge sharing. The CWG aims to fulfil the following objectives:

1. To enable the process evaluation teams to better understand how practitioners are delivering all components of the intervention, providing insight into the nature of the intervention and its delivery in practice in order to contextualise pilot research findings and inform the design of the process evaluation used in the trial
2. To enable practitioners to share knowledge with each other in order to benefit from cross-site learning and offer reciprocal advice based on what is going well and what is proving to be challenging in delivering the new intervention in each study centre
3. To enable practitioners to play a crucial role in shaping the ongoing research process by giving them opportunities to suggest topics or questions for exploration in data collection and analysis, thereby enhancing the sensitivity and rigour of the research
4. To collaboratively identify and action changes and refinements to the intervention between each wave of the pilot phase and to finalise the design of the intervention before the larger scale trial

### The underpinning ethos of the CWG process: mobilising knowledge in partnership

The CWG process is presented as a knowledge mobilisation approach, which recognises and respects the equality and necessity of different types of knowledge. Unlike the outdated term ‘knowledge translation’—which harks back to traditional hierarchies of evidence and implies that knowledge use is linear—the concept of ‘knowledge mobilisation’ recognises that knowledge is varied and multi-directional [2]. The CWG process treats research evidence as *one* particular knowledge type that, in combination with other forms of knowledge, can shape and determine best practice [3–5]. In DAFNE*plus*, we view the pilot phase as an opportunity to co-create the intervention by weaving together knowledge generated through the process evaluation with practitioner experience and expertise. Many of the practitioners involved in DAFNE*plus* are co-applicants/collaborators on the programme grant and, as such, are active contributors not only in designing and delivering the intervention but also in reflecting on and responding to emerging research findings. This democratic, iterative, co-design process is also underpinned by the principles of participatory action research, which seeks to generate actionable knowledge and practical outcomes in collaboration with—rather than for—practitioners [5, 6]. All process evaluations are informed by theory—whether formal or informal—and practitioners have and develop their own personal theories about what works best and why. The CWG process seeks to harness this combined knowledge as a means of developing optimal interventions and informing sensitive research designs.

### Creating a structure for reflective and responsive dialogue: the right knowledge, at the right time

The CWG process combines a strategic mix of monthly teleconferences and face-to-face meetings. The purpose of the face-to-face meetings is to agree upon definitive changes to the intervention, while the monthly teleconferences serve as a real-time sharing platform for regular updates and highlighting emerging issues as they arise. To encourage reflection, provide a focus for discussion and promote clear decision-making, all meetings will draw upon Borton’s reflective prompt questions: ‘What?’, ‘So What?’ and ‘Now What?’ [7]. This enables reflection on cumulative knowledge about the following:

- What?—A discussion about what has been going well and not so well with the content, delivery and receipt of the intervention
- So what?—A discussion around the implications for both research design and intervention refinement
- Now what?—An agreement about what needs to happen next

An operational description of both meeting formats (teleconference and face-to-face) is provided next.

#### Monthly teleconferences

The aim of the teleconferences is to highlight early in the process which elements of the intervention may potentially need to change and identify what further information is needed to help inform this decision-making at the face-to-face meetings. Practitioners share reflections on their successes and challenges when implementing the intervention, while the researchers share findings and reflections arising from ongoing data collection and preliminary analysis. Borton’s questions will be operationalised using a discussion matrix we designed (see Table 2 for the matrix template and notes on completion). Approximately 1 week before each teleconference, the Chair will invite all CWG members to email content for the ‘what’ column of the matrix. The Chair will summarise this feedback into easily digestible, bullet point format and organise the information thematically underneath each intervention component in the matrix. Based on the project timeline, and any indication given by the CWG members before the meeting, the Chair will structure the matrix in order of priority, with the most pressing topics for discussion at the top. In the ‘so what’ column, the Chair will identify prompt questions, to help focus discussion. During the meeting, the Chair will guide the discussion through each row in the matrix, coming to a decision about what needs to happen next in order to address each of the emerging issues, either with implications for intervention delivery (e.g. changes to the intervention) or research design (e.g. adding new questions to the interview topic guide). After the meeting, all actions will be noted in the ‘now what’ column of the reflective matrix and distributed to the CWG—alongside a traditionally detailed minutes—after the meeting.

**Table 2:** Template for CWG teleconference discussion matrix

WHAT… is going well/ not going as well?	SO WHAT… does this mean for (a) intervention delivery and redesign (b) the research process?	NOW WHAT… do we recommend needs to be done, by who, when?
**COURSE**		
**ᅟ**	**ᅟ**	**ᅟ**
**FOLLOW-UP**		
**ᅟ**	**ᅟ**	**ᅟ**
**TECHNOLOGY**		
**ᅟ**	**ᅟ**	**ᅟ**
**Notes on Completion for the Chair**		
*Invite CWG members to email feedback one week before the teleconference date. Summarise feedback and populate this column prior to each meeting - condense information into bullet point format so it is quick to read but still thorough and representative. Add as many rows as needed to organise discussion points into broad topics under each of the main intervention components. Prioritise the order in which intervention components are discussed and adapt the matrix structure each month with the highest priority component at the top. Priority can be established based on the project timeline and CWG members’ feedback.*	*Prior to each meeting, populate this column with prompt questions designed to explore the information in the ‘what’ summary. Tailor these questions to the specific issues arising. These questions should be chosen to help progress discussion towards a decision in the ‘now what’ column. Examples include: ‘do we need to consider changing this?’ ‘What further information do we need before we can make a decision?’ ‘What impact would a change have on the intervention and/or research process as a whole?’ During the meeting, additional questions and discussion points are likely to arise organically and these should be added to this column afterwards to keep a record of the decision making process.*	*Populate this column after each meeting with a list of the agreed actions, deadlines and person(s) responsible. It is important to note that actions will not necessarily be changes to the intervention. Actions, for example, may be a decision to seek more information for the next meeting (*e.g. *by adding questions to interview topic guides or asking facilitators to reflect on a specific area of delivery in supervision), or a decision to wait until the next face to face meeting before agreeing any course of action.*

#### Face-to-face meetings

The aim of the face-to-face meetings is to make definitive decisions about intervention design. These full-day meetings will mirror the structure of the teleconferences but, rather than using a single matrix, detailed matrices will be used for each aspect of the intervention. These matrices will be prepared by the Chair and the study manager in advance of each face-to-face meeting. The ‘what’ column will be populated with a combined summary of the findings from the process evaluation, patient advisory group meeting minutes and the cumulative learning from the monthly teleconferences which draws upon practitioner experience and expertise. This combined knowledge will be grouped thematically into as many matrices as needed (e.g. one matrix for each session of the course curriculum) and prepared in advance with ‘so what’ prompt questions to guide discussion. Using progressive rounds of small group discussion (each group comprising at least one researcher, clinical psychologist, a practitioner and a physician) and plenary feedback sessions, the day-long meeting should culminate in a set of collectively agreed changes to the intervention. Each group will update their individual matrices with details of their discussions (so what) and agreed actions (now what) and will give these to the Chair for typing up after the meeting. A complete set of matrices plus a summary report of the agreed actions will then be distributed by the Chair within a week of the face-to-face meeting.

#### A note on timing

**Fig. 2:**
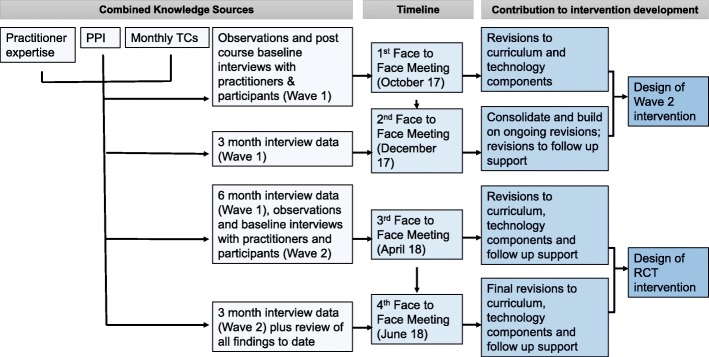
Timetable of strategically timed CWG face-to-face meetings in DAFNE*plus*. Legend: the timing of each face-to-face meeting showing the different knowledge sources feeding into each meeting and the corresponding outcomes

In DAFNE*plus*, monthly teleconferences will commence at the start of pilot delivery in wave one and will continue until the end of the intervention development stage. Two face-to-face meetings will take place between waves one and two, with a further two face-to-face meetings between wave two and the trial. The meetings are timed in accordance with the availability of data from the process evaluation, permitting sufficient time for data collection and analysis to inform development of the intervention in stages. This is depicted diagrammatically in Fig. 2.

Staggering the face-to-face meetings is intended to accommodate the large amount of information to be processed within a feasible timescale and to facilitate prioritisation. Putting a structured timeline in place, with clearly identified periods for implementing changes, will be important to prevent reactionary, ad hoc changes to the intervention during the pilot phase and to ensure that all changes are evidence based and efficient. Decisions will thus be based on cumulative knowledge and will wait for participant feedback via the process evaluation rather than being pre-emptive and opinion based. Moreover, intervention refinements will be actioned only once there is sufficient agreement on the basis of all available information.

### CWG membership: drawing on different types of knowledge

The CWG operates as a knowledge-sharing platform, providing a series of fixed points for cross-fertilisation of learning between an inter-disciplinary team. Where researchers and practitioners work separately on relatively discrete parts of the research programme, across different work streams and with different roles and responsibilities, the CWG provides an opportunity to share higher level learning. In DAFNE*plus*, the CWG will involve 25 members of the research programme team, including the practitioners delivering the intervention (predominantly nurses and dietitians with some input from physicians), the clinical psychologists inputting to intervention design and providing practitioner supervision, the research teams conducting the process evaluation (a team of behavioural psychologists and a team of social scientists), the study manager and the Chief Investigator. In their site-specific supervision meetings (comprising a phone call approximately 1 week before and mid-way through each course, alongside a weekly email contact), practitioners have the opportunity to confidentially consolidate their understandings of the successes and challenges of intervention delivery, before distilling the key lessons that need to be shared across sites and disciplines at the CWG meetings. Having led the design of the CWG process, the first author (one of the social scientists conducting the process evaluation) will act as the CWG Chair. The study administrator will also work as a key member of the CWG, providing support with logistics and taking detailed minutes from each meeting. The role of the different members of the CWG are summarised in Table 3.

**Table 3:** Membership and roles of the CWG

Category	*n*	Role
Practitioners delivering intervention	12	Intervention design and delivery—led by DAFNE-trained dietician and nurse educators, with input from DAFNE-trained physicians (3 NHS centres).
Diabetes specialist dieticians	4	
Diabetes specialist nurses	5	
Consultant diabetologists	3	
Clinical psychologists	2	Intervention design and provision of clinical supervision to DAFNE practitioners delivering the intervention.
Process evaluation research teams	6	Behavioural psychologists: intervention design and process evaluation, via post-course patient and practitioner interviews, observation and fidelity assessment. Social scientists: process evaluation via longitudinal interviews at 3 and 6 months.
Behavioural psychologists	4	
Social scientists	3^a^	
Study manager	2^b^	Oversight of DAFNE*plus* research programme (project management, ethics, governance etc.) with expertise in knowledge mobilisation and shared facilitation of face-to-face meetings.
CWG Chair	1	Responsible for chairing the CWG meetings and delivery of CWG processes. Shared facilitation of face-to-face meetings with the study manager. Member of the social science process evaluation team with expertise in knowledge mobilisation.
CWG Administrator	1	Administrative support to CWG meetings (minute taking, room booking, organisation of meetings and travel).
Chief Investigator	1	Leadership of DAFNE*plus* Programme Grant and research-active Consultant Diabetologist.
Total	25	

^a^One of the social scientists is also the CWG Chair. They are included in both sections of the table to highlight the dual role but are only counted once in the final total

^b^A new study manager was appointed in March 2018 but the previous incumbent maintained their role in CWG

### Involving patient advisory groups in the CWG: working collaboratively and reflexively to incorporate lived knowledge in iterative intervention design

Patient and public involvement (PPI) is important for developing interventions that are tailored to the contexts in which people manage their own health, encouraging research teams to think beyond their current ways of knowing and building strong relationships with key influencers in the community to facilitate dissemination and impact [8]. The CWG process adopts a collaborative approach to PPI, which is defined by the NIHR (p. 22) as ‘working more closely with members of the public, returning to ask them for further information, and developing an ongoing relationship with them’ [9]. This entails a two-way flow of knowledge, where patient advisory groups will be updated routinely about pilot intervention delivery and asked to make recommendations for intervention refinements to be fed directly into CWG decision-making. To operationalise this process, patient advisory groups will mirror the CWG discussion process by working through a matrix prepared by the CWG Chair in collaboration with the patient advisory group Chair. The matrix will summarise the issues arising in intervention delivery derived from interviews with facilitators and participants in the process evaluation (what) and will seek the patient advisory groups’ expert opinions on the issues arising (so what) and their recommendations for how the CWG refine the intervention (now what). The recommendations of the patient advisory group will then provide a key knowledge source for the CWG meetings.

In turn, decisions made at the CWG meetings will be reported back to the patient advisory groups to elicit their reflections on the usefulness and feasibility of the actions identified and highlight where further refinement might be needed. This report will ensure that the CWG are accountable to the patient advisory group by providing feedback about how their recommendations are being acted upon. Using this iterative and dynamic—as opposed to one-off and static—approach to PPI permits mutual contribution of knowledge as the patient advisory groups are able to actively inform new ideas for intervention development, not simply commenting on relatively unchangeable decisions that are pre-identified by ‘experts’. A collaborative approach will be key to overcoming the all too common frustrations reported by patient and public partners who feel that they are not always listened to, they perceive that expert views are given priority over their own and they rarely receive feedback about if and how their input has been acted upon [8].

### How will we know if the process works and what lessons can we share with others?

A qualitative study has been built in to this process to understand how the CWG works and how its processes and procedures might be improved for use in future pre-trial pilots and intervention development studies. The aim of this sub-study is to evaluate the CWG and shed light on how CWG communication occurs between the practitioners delivering the DAFNE*plus* intervention and the researchers conducting the process evaluation, in order to distil transferable lessons about how to best operationalise iterative intervention design. This will also include an exploration of the acceptability and feasibility of the CWG process, eliciting members’ views on the logistical and practical aspects of taking part (such as the frequency, timing and format of meetings) to learn lessons about how to optimise engagement within future teams wishing to use this process.

#### Research questions

The qualitative study addresses the following research questions:

1. How did the CWG facilitate decisions about intervention refinement?
2. What were the opportunities and challenges posed by the CWG process?
3. What were researchers’ and practitioners’ experiences of, and views about, taking part in the CWG process?
4. How could the CWG process be improved?

#### Participants

All members (*n* = 25) of the DAFNE*plus* programme grant team participating in any of the teleconferences and face-to-face meetings will be invited to take part. They will be contacted by email and provided with a participant information sheet and consent form. They will be asked to provide a blanket consent to take part in the study, with clear opportunities to withdraw from the study (or from specific parts of the study) at any point in the process without giving a reason. Other members of the wider programme grant team, who may join the teleconferences or face-to-face meetings on an ad hoc basis, will also be informed of the study and invited to give informed consent using the same process.

#### Data collection

Multiple forms of data will be collected to provide a detailed account of the CWG process. All meetings will be audio-recorded, and all documentation (such as, agendas, minutes, decision matrices and emails relating to the meetings) will be used in analysis. Mirroring the inputs into the CWG, the first author will make written field notes during and after the meetings to capture interactions and to note down reflections about what worked well and not so well during each meeting. At the end of the second and fourth face-to-face meetings, CWG members will be invited to take part in a focus group lasting approximately 1 h to elicit their experiences of and views about participating in the CWG. The focus group will be conducted by a researcher independent to the DAFNE*plus* study team and will explore participants’ views about the frequency, method, timing and impact of the meetings and how, if at all, communication and collaborative working might be improved. Participants who are unable to attend the focus group sessions or wish to offer individual feedback will be offered an opportunity to express their views via email or in a telephone interview.

#### Data analysis

Audio recordings from the teleconferences and face-to-face meetings will be transcribed and analysed using a discourse analysis approach [10] to explore how decisions about intervention redesign were made, track the progression of decision-making over time and explore the social processes and dynamics shaping decision processes. Meeting documentation will be analysed by drawing on the principles and processes of documentary analysis outlined by Jacobsson [11] to identify attendance patterns across participants and if, how and why attendance fluctuated at different time points in the study; to trace decisions as they are made and refined over time; and to explore how documents shape interactions and are co-created in use. This will enable a longitudinal exploration of the decision processes involved and help us to establish whether and how initial ideas might have differed to the final decisions made about intervention refinement. Finally, focus group and interview transcripts, and data from emails, will be analysed thematically [12], to elicit participants’ experiences of and views about taking part in the CWG. Conducting the focus groups at two time points will enable exploration of whether, and how, participants’ views have changed over time. This approach to triangulating different data sources and analytic approaches [13] mirrors the dynamic knowledge mobilisation ethos underpinning the CWG process, affording rich, multi-level insights into how the CWG process operates and creating multiple opportunities for identifying areas for improvement.

#### Ethical considerations

Ethical approval for the in-built qualitative study has been granted by the Usher Research Ethics Group at the University of Edinburgh. Owing to the small numbers involved in the CWG and the very distinct professional roles of participating individuals, however, assurances of full anonymity are not possible. However, participants will be made aware of this prior to consenting and will have the opportunity to review all outputs prior to dissemination to approve the content and request rewording or removal of text relating to themselves if necessary.

## Discussion

This paper outlines a novel structured process for collaborative intervention development. It is presented here as a potential blueprint for supporting iterative feedback between researchers and practitioners to facilitate a focused, shared decision-making in a pre-trial and in intervention development studies. Although we have recognised that ‘knowledge mobilisation’ is now defined as a social, dynamic process, in practice, there is still a tendency to only think about knowledge mobilisation after research completion (that is, using traditional forms of dissemination) rather than within the relational context of an ongoing research programme [14]. Moreover, much existing knowledge mobilisation literature is typically founded on the premise that researchers and practitioners operate in different spaces, whereby researchers ‘push’ their research into the world of practice and practitioners ‘pull’ in useful learning from research to support and change practice [4]. This is counter to the requirements outlined in the MRC guidance [1] for pre-trial work and, in the context of the DAFNE*plus* pilot study, where practitioners and researchers are operating together as equal collaborators in contributing to both intervention and research design pre-trial, the circumstances are quite different.

In order to support effective, collaborative communication practices at this stage of intervention development and prior to a trial, there is a need for novel approaches that draw on and extend beyond existing lessons from the world of knowledge mobilisation. To date, there has been little attention given to practitioners ‘pushing’ evidence (determining research agendas based on problems in practice) and researchers ‘pulling’ knowledge from practitioners (seeking input into research ideas and processes). Although a plethora of knowledge mobilisation models exists—a comprehensive review identified no less than 18 different types [3]—it is notable that none provide practical, step by step guidance on how to operationalise effective knowledge mobilisation and very few have been empirically tested or evaluated in different settings [3]. The CWG process attempts to address these gaps by providing structured, operational guidance on how to mobilise knowledge for intervention development, and conducting a qualitative study to elicit tangible and transferable lessons from the DAFNE*plus* experience. At the time of submitting this paper, we have just commenced wave two of the DAFNE*plus* pilot study. We held the first CWG teleconference on June 27, 2017, and to date, we have held nine teleconferences and three face-to-face CWG meetings. Ethical approval for the sub-study was granted on August 22, 2017 (reference: 1732) and, so far, we have undertaken one focus group as well as individual interviews with two members of the study team. We present the CWG process here as a potential solution to address a gap in the intervention development literature. By developing and testing out a new approach to knowledge mobilisation within the context of the DAFNE*plus* pilot study prior to an RCT, we hope to advance the evidence base for an under-explored knowledge mobilisation context.

## Abbreviations

CWG: Collaborative Working Group
DAFNE/DAFNE*plus*: Dose Adjustment for Normal Eating
MRC: Medical Research Council
NHS: National Health Service
NIHR: National Institute for Health Research
PPI: Patient and public involvement
RCT: Randomised control trial
